# Intense FDG Uptake in the Common Bile Duct Post-ERCP Mimics Acute Infectious Cholangitis

**DOI:** 10.3390/tomography8060248

**Published:** 2022-12-18

**Authors:** Neel P. Mistry, Wanzhen Zeng

**Affiliations:** 1 Faculty of Medicine, University of Ottawa, Ottawa, ON K1H 8M5, Canada; 2 Division of Nuclear Medicine, Department of Medicine, The Ottawa Hospital, Ottawa, ON K1Y 4E9, Canada

**Keywords:** cholangitis, [^18^F] FDG PET, ERCP

## Abstract

In patients with obstructive pancreatitis due to choledocholithiasis, endoscopic retrograde cholangiopancreatography (ERCP) is the standard of care. ERCP-induced inflammation or infection of the common bile duct (i.e., cholangitis) is a rare complication with a high mortality rate in severe cases. We report an unusual case of incidental findings of intense FDG uptake in the common bile duct one month post-ERCP without clinical features of acute cholangitis, indicative of inflammation of CBD associated with or exaggerated by ERCP.

## 1. Introduction

Endoscopic retrograde cholangiopancreatography (ERCP)-induced cholangitis is rare, with a rate of 1% or less [1,2], but it could be serious in severe situations. It is thought to result from enteric bacteria entering the biliary tree via the hematogenous route or following endoscopic or radiologic manipulation. Patients typically present with fever, jaundice, and abdominal pain, but hypotension and altered mental status can ensue in severe cases. Significant risk factors in univariate analysis include percutaneous endoscopic procedures, stenting of malignant strictures, and failed biliary access or drainage [1,2,3].

We present a case of intense fluorodeoxyglucose (FDG) uptake in the common bile duct (CBD) one month post-ERCP in a patient who initially presented with acute pancreatitis.

## 2. Case Report

A 67-year-old male with a remote history of colon cancer post-proctocolectomy and cholecystectomy presented with a 24 h history of abdominal pain radiating to the back. He felt generally unwell and denied any fever or chills. The patient had prior ERCP.

**Figure 1:**
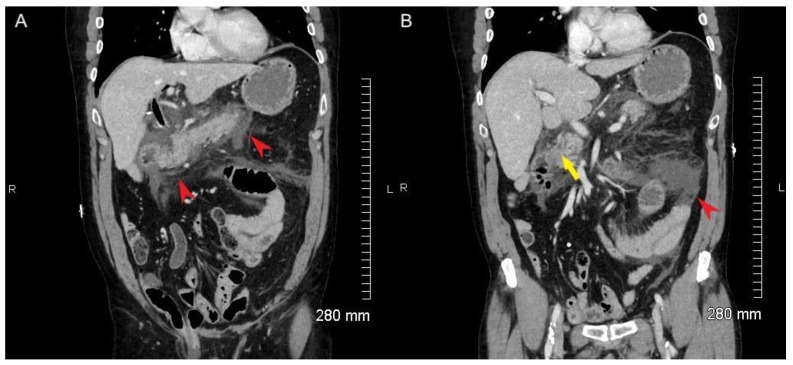
CT abdomen and pelvis with contrast demonstrates significant peripancreatic fluid and fat stranding extending to porta hepatis, consistent with acute pancreatitis (**A**,**B**, red arrowheads). The patient is status post cholecystectomy. There is pneumobilia, likely associated with prior cholecystectomy. The biliary tree is prominent with abnormal wall enhancement. The CBD measures 9 mm proximally and 5 mm distally. No definite signs of choledocholithiasis are seen in the distal CBD (**B**, yellow arrowhead).

Initial laboratory testing showed critically elevated lipase and elevated white blood cell count with low sodium. ALP, ALT, AST, and bilirubin were normal. There was no evidence of bacteremia or sepsis. Initial ultrasound was equivocal for gallstones. Initial CT of the abdomen and pelvis demonstrated peripancreatic fat stranding and heterogenous appearance of the parenchyma consistent with acute pancreatitis (Figure 1, red arrowheads) but no definite choledocholithiasis. The CBD measured 8 mm with slight mural thickening. The biliary tree was prominent with abnormal wall enhancement, but no definite signs of choledocholithiasis were noted in the CBD (Figure 1B, yellow arrowhead). A repeat biliary ultrasound demonstrated sludge and choledocholithiasis in the CBD.

The patient subsequently had ERCP with removal of sludge and stone materials from the CBD. Within 24 h of ERCP, the patient’s pancreatitis improved with supportive management, with improvement of abdominal pain and resolution of leukocytosis. The patient was discharged 2 days post-ERCP.

**Figure 2:**
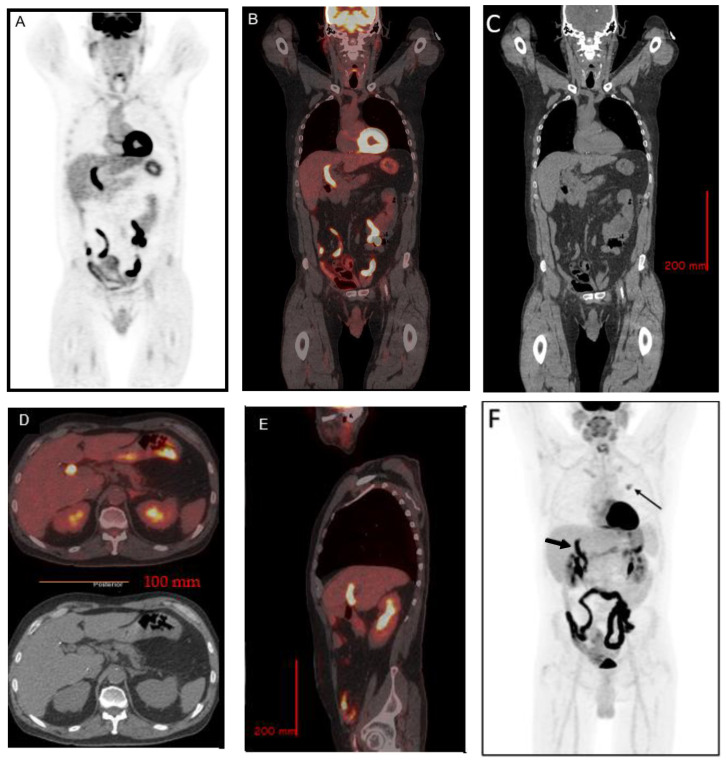
Intense ^18^F-FDG uptake (SUV_max_ = 13.3) along the CBD (coronal images, (**A**). PET, (**B**). PET/CT, (**C**). CT, (**D**). Trans-axial images, PET/CT and CT, (**E**). Sagittal images, PET/CT and (**F**). Maximum intensity projection, thought to be due to ERCP-induced inflammation. There is mild fat stranding adjacent to the CBD on the corresponding low-dose CT images without a definite radiopaque stone in the distal CBD. On MIP, intense uptake at common bile duct is noted (short arrow). There is a hypermetabolic lesion in the left lung (long arrow), confirmed to be a metastasis from colon cancer on biopsy. The patient has a remote history of colon cancer 26 years ago, status post-colectomy with ileostomy. Intense uptake in the bowel loops is secondary to metformin use.

**Figure 3:**
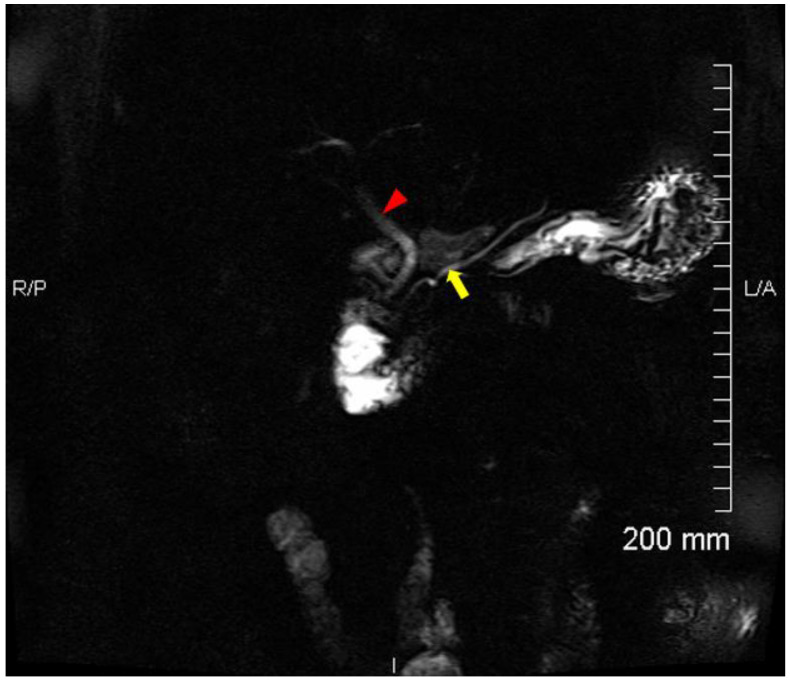
A follow-up MRCP 9 days after the ^18^F-FDG PET shows no evidence of acute cholangitis or acute pancreatitis. Coronal T2 weighted MIP imaging (SSTSE, slice thickness 30 mm) shows the CBD (arrow head) and main pancreatic duct (arrow). No lesions or abnormal findings within the CBD. The size of the CBD measures 6 mm.

Due to incidental CT findings of a left upper lobe pulmonary nodule, an [^18^F] FDG positron emission tomography (PET) was performed approximately one month post-ERCP. In addition to a moderately hypermetabolic left upper lobe pulmonary nodule, which was later proven to be a metastasis from colon cancer, there were incidental findings of increased uptake in the CBD, with a SUV_max_ of 13.3, suspicious for cholangitis (Figure 2). A follow-up magnetic resonance cholangiopancreatography (MRCP) was performed 9 days after the [^18^F] FDG PET showed no evidence of cholangitis, pancreatitis, or choledocholithiasis (Figure 3).

## 3. Discussion

We reported a case of intense FDG uptake in the CBD in a patient one month post-ERCP.

Cholangitis is an inflammation of the bile duct. Acute cholangitis occurs most commonly from bacterial infection of the bile ducts, characterized by fever, jaundice, and abdominal pain (Charcot’s triad), which in most cases is a consequence of biliary obstruction [4]. Diagnosis is commonly made by the presence of clinical features, laboratory tests, and imaging studies. At the time of [^18^F] FDG PET imaging, there were no clinical features suggestive of acute infectious cholangitis based on Charcot’s or the 2018 Tokyo criteria [5,6]. In addition, a follow-up MRCP 9 days post-[^18^F] FDG PET showed no evidence of cholangitis.

Acute cholangitis could co-exist with acute pancreatitis. In a single-center study, 32 (23%) patients presented with both conditions over 10 years (the diagnosis of acute cholangitis was based on Charcot’s triad), and the majority were treated with ERCP [7]. It is possible that the patient may have had acute cholangitis at the time of acute pancreatitis before ERCP, although it is unlikely that the patient had infectious or severe cholangitis, as there was no evidence of fever, chills, jaundice, or abnormal liver function tests. In addition, there was no significant CBD dilation on CT. During the hospital stay, no diagnosis of acute cholangitis was made.

Increased uptake in the CBD associated with malignant and benign neoplasm has been reported. Cholangiocarcinoma and invasive intraductal papillary neoplasm of the bile duct are FDG avid [8,9,10]. FDG uptake in the CBD has been reported in rare tumors, such as malignant intraductal papillary mucinous neoplasm of the bile ducts [11], tubular adenoma of the CBD [12], and biliary papillomatosis [13]. It has also been reported in CBD tuberculosis [14] and bile duct thrombosis associated with hepatocellular carcinoma [15].

[^18^F] FDG PET is a molecular imaging modality commonly indicative of various cancers. It is also indicated in identifying the cause of fever and inflammation of unknown origin [16,17,18]. Focal FDG uptake secondary to stent placement has been reported [19,20]. Nagasaki et al. [19] reported high FDG uptake at the site of biliary stent in a patient with pancreatic cancer, suspected to be related to focal inflammation caused by insertion of the metallic stent.

In our center, FDG PET is only performed for oncology indication. In this patient with a solitary lung lesion referred for FDG PET with incidental findings of CBD uptake post-ERCP, there was no indication for FDG PET follow-up. The etiology of the intense FDG uptake is speculative, based on the available radiological and FDG PET imaging, as well as clinical follow-up. The intense FDG uptake could possibly be due to CBD smooth muscle peristaltic spasm, although no such findings have been documented. The incidental findings of intense FDG uptake in the CBD in our case could be representative of a resolving inflammatory process due to ERCP or exaggerated by ERCP, supported by the patient’s lack of symptoms and normal follow-up MRCP. This is different from typical acute cholangitis caused by introduction of enteric bacteria through a contaminated endoscope.

## 4. Conclusions

Increased FDG uptake along the CBD in patients post-ERCP could be due to an inflammatory process caused or exaggerated by ERCP. This should be distinguished from acute infectious cholangitis, as the two have different etiology and clinical presentation.

## Data Availability

Not applicable.

## Abbreviations

ALP—Alkaline Phosphatase; ALT—Alanine Transaminase; AST—Aspartate Transaminase; CBD—Common Bile Duct; CT—Computed Tomography; ERCP—Endoscopic Retrograde Cholangiopancreatography; [^18^F] FDG PET—^18^F-Fluorodeoxyglucose Positron Emission Tomography; MRI—Magnetic Resonance Imaging; WBC—White Blood Cell.
